# A Chinese Family with Familial Dysalbuminemic Hyperthyroxinemia (FDH) due to R242H Mutation on Human Albumin Gene: Reevaluating the Role of FDH in Patients with Asymptomatic Hyperthyroxinemia

**DOI:** 10.1155/2019/5947415

**Published:** 2019-09-09

**Authors:** Hongbing Liu, Jianmin Ran, Chuping Chen, Guangshu Chen, Ping Zhu, Rongshao Tan, Yan Liu

**Affiliations:** ^1^Endocrinology Department, Guangzhou Red Cross Hospital, Medical College of Jinan University, Guangzhou 510220, China; ^2^Institute of Diseases-Oriented Nutrition Research, Guangzhou Red Cross Hospital, Medical College of Jinan University, Guangzhou 510220, China

## Abstract

**Objective:**

Familial dysalbuminemic hyperthyroxinemia (FDH) has now become an established cause for spurious asymptomatic hyperthyroxinemia. Several different codon mutations on albumin gene had been identified. We here provided an established but rarely reported heterozygous mutation based on gene sequencing results from a Chinese family.

**Methods:**

The proband is a 14-year-old girl with light goiter and asymptomatic clinical presentations, whose thyroid function test by a one-step immunoassay showed increased free thyroxine (FT4) and free triiodothyronine (FT3) but nonsuppressed thyrotropin (TSH). All thyroid auto-antibodies were in the normal range. Blood samples were collected from her and most of her immediate family members for target gene sequencing and verification.

**Results:**

Hyperthyroxinemia was also confirmed in the proband's mother and one of her uncles and his son. In the proband and these three pedigrees, the high-throughput gene screening sequencing and the following Sanger sequencing disclosed a heterozygous mutation in the albumin gene, which located in its exon 7 (c.725G > A), and correspondingly leads to an arginine replacement with a histidine (R242H) in its protein. This is an established mutation named as R218H if present without signal peptide sequence.

**Conclusions:**

For patients with asymptomatic hyperthyroxinemia, FDH should be clinically excluded before embarking on further investigations for other specific causes.

## 1. Introduction

Familial dysalbuminemic hyperthyroxinemia (FDH) was reported in the first place by Henneman et al. [1] and Lee et al. [2] in 1979. It has been now confirmed as a congenital benign variant with an autosomal dominant inheritance mode [3]. FDH's incidence rate is about 0.2% in common populations [4], and hence is a main cause for asymptomatic hyperthyroxinemia [5]. Most probands with FDH are incidentally identified by the laboratory test of serum thyroid indexes, which characterized by increased total thyroxine (TT4) and total triiodothyronine (TT3), but usually with nonsuppressed thyrotropin (TSH) [5]. Serum concentrations of free thyroxine (FT4), free triiodothyronine (FT3), may present falsely elevated by using routine one- or two-step immunoassays [6], a condition being similar with asymptomatic hyperthyroxinemia caused by abnormalities of other thyroxine-binding proteins such as thyroxine-binding globulin (TBG) [7] or thyroxine-binding prealbumin (TBPA) [8]. Subjects with FDH are mostly free of thyrotoxic symptoms [5], but there is a risk that patients can be offered inappropriate treatment such as antithyroid drugs (ATD) or ^131^iodine radiation [9]. To avoid these unnecessary therapies, more and more strategies, including appropriate laboratory methods and genetic sequencing should be clinically adopted to identify FDH patients.

Various codon mutations of the albumin gene result in abnormal human serum albumin (HSA) with increased binding affinity to thyroxine (T4) and/or triiodothyronine (T3), named as FDH-T4 and FDH-T3, respectively [5, 10]. The first reported mutation identified by Sunthornthepvarakul et al. [11] in 1994 was an arginine-to-histidine substitution at the residue 218 (R218H) of albumin (R242H if with the sequence of its signal peptide), and so far, the mutation is the most common type reported in FDH families from Caucasians [12], Hispanic/Puerto Rican [13], Brazilian [14], and Chinese [15–18]. The mutation leads to modestly increased serum TT4 and lightly increased TT3 [12–18]. The second type, an arginine-to-proline mutation (R218P), with significantly elevated TT4, has been mostly descripted in Japanese [19, 20] and Swiss families [21]. Other rare mutations include R218S from Bangladeshi [22] and R222I from Somali and Croatian [23]. Especially, the L66P mutation from a Thai kindred [24] shows remarkably increased TT3.

We here report a heterozygous mutation of the albumin gene, with replacement of arginine by histidine at codon 242 (R242H) in a Chinese family. The replacement resulted in nearly 2.2-fold and 1.5-fold elevations of serum TT4 and TT3, respectively, in all affected family members; simultaneously, FT4 and FT3 mildly increased in the proband and several other affected relatives. In Chinese, we believe that this will be the fourth family related to the same codon mutation of the human albumin gene according to a comprehensive document retrieval including published Chinese documents [15–18].

## 2. Patients and Methods

### 2.1. Patients

The proband is a 14-year-old girl who was born to unrelated Chinese parents and lives in Henan province, China. In a routine physical examination on 2017, she was diagnosed with hyperthyroidism owing to a slight goiter and the elevated serum FT4 but normal TSH (exact results are not available). She did not undergo any specific symptoms related to thyrotoxicosis, and her body weight remained stable during the last 3 months. She was prescribed with methimazole (10 mg/d) in the local hospital mainly because of her simultaneous goiter and elevated serum thyroxine, irrespective of the nonsuppressed TSH. Fortunately, the treatment was discontinued one month later because of her aggravated goiter and tiredness. Two months later, she came to our attention at Guangzhou Red Cross Hospital for a further evaluation. A routine physical examination was conducted, and exactly slightly enlarged thyroid was found, but without any other obvious signs such as proptosis, resting tachycardia, and hand tremor. Serum thyroid function (one-step immunoassay) was repeated and listed as follows: FT3 6.71 pmol/l (reference range 3.1–6.8 nmol/l), FT4 34.58 pmol/l (reference range 12.0–22.0 pmol/l), simultaneous TSH 1.38 mIU/l (reference range 0.27–4.20 mIU/l), and thyroid auto-antibodies including antithyroid peroxidase antibody (TPOAb), antithyroglobulin antibody (TGAb), as well as anti-TSH receptor antibody (TRAb) were all in normal range. An ultrasound detection of the thyroid also showed no significant morphological abnormalities in the proband girl. Finally, no treatment was administered for her asymptomatic presence.

One year later in 2018, the girl still seemed healthy without any clinical complaints. The results of blood cell counts and biochemical measurements were almost fallen into their normal ranges. A whole serum thyroid function test (the same method as mentioned above) was performed, and the results disclosed not only elevated serum FT4 and FT3 but also remarkably elevated serum TT4 and TT3. All exact data are listed here as follows: FT3 7.30 pmol/l, FT4 32.08 pmol/l, TSH 1.34 mIU/l, TT4 391.4 nmol/l (reference range 66.0–181.0 nmol/l), and TT3 8.9 nmol/l (reference range 1.3–3.1 nmol/l). Thyroid auto-antibodies mentioned above remained normal, and ultrasound showed no further morphological deterioration. To explore the actual reason of her hyperthyroxinemia, in the first place, we did not realize that the laboratory results might be interfered by other factors; pituitary MRI was performed, and no signs of tumor were shown. Simultaneously, we did exons sequencing for the thyroid hormone receptor *β* (TR*β*) gene and also got negative results. The latter two tests obviously excluded pituitary TSH adenoma and resistance to the thyroid hormone (RTH), two diseases also characterized by elevated thyroxines and probably normal TSH.

The proband's mother, aged 40, also exhibited similar results of thyroid function in a routine physical examination in 2017. Her results are listed as follows: FT3 5.61 pmol/l, FT4 28.26 pmol/l, and TSH 2.19 mIU/l (reference ranges are the same as the above described). She had been diagnosed with subacute thyroiditis 5 years ago but cured finally. No specific symptoms exist in her now. One of the proband's uncles were described as “high level of serum thyroxine” by local doctors 5 years ago (data not available). There is no other positive history of hereditary diseases in this family.

### 2.2. Samples

The proband and her immediate family members were sampled at a fasting day, and blood samples were assigned for regular biochemistry assays, thyroid function testing, and gene sequencing. All family members enrolled in the investigation signed the written consent for genetic testing. No members were taking antithyroid drugs, L-thyroxine, or other specific agents which might affect the blood concentration of thyroxine hormones. All enrolled members were free of biotin-contained agents which were believed to interfere with thyroxine hormone measurement by using the biotin-avidin method [25].

### 2.3. Biochemical Assays

Serum concentrations of alanine aminotransferase (ALT), aspartate aminotransferase (AST), albumin (ALB), and creatinine (CREA) were measured by enzymatic methods on an automatic biochemical machine (Hitachi 7600, Japan). These parameters were measured to exclude the possible effects of abnormal hepatic and renal function on laboratory results.

### 2.4. Thyroid Function

Serum concentrations of TT4, TT3, FT4, FT3, and TSH were measured on a one-step electro-chemiluminescence immunoassay analyzer (ECLIA, Elecsys analyzer, Roche Diagnostics, Tokyo, Japan) following the operating instructions. Serum thyroid auto-antibodies including TGAb, TPOAb, and TRAb were also assayed on the same system by using the corresponding kits (Roche Cobas e 411 analyzer, Tokyo, Japan).

### 2.5. Gene Sequencing

Because of a negative TR*β* sequencing in the proband, the extracted DNA of the proband was amplified and purified by the TruSight One Sequencing Panel (Illumina Inc., USA); the final genomic library was set up and then sequenced in the Illumina MiSeq System (Illumina Inc., USA) by using the next-generation sequencing (NGS) technique [26]. The positive codon mutation of the albumin gene was verified by the Sanger sequencing, which has been the “golden standard” of gene mutation analyses [27]. As for the other immediate family members after the proband, the extracted DNA was only analyzed and confirmed for the same codon mutation by the direct Sanger sequencing. Specific primers used for the Sanger sequencing are as follows: *forward*, TGTTGCCCCTTTTAGAGTTCCT and *reverse*, TCAACCCACTGTCAGCTATCA, respectively.

## 3. Results

**Figure 1:**
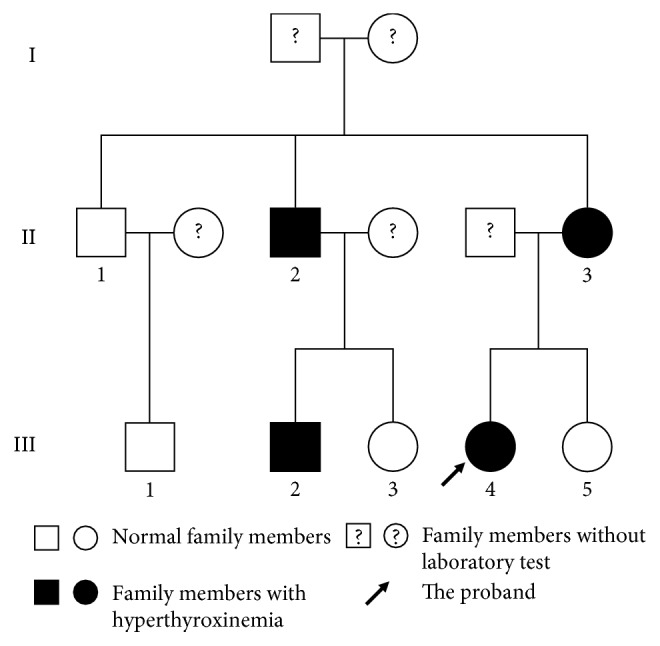
The pedigree of a Chinese family with familial dysalbuminemic hyperthyroxinemia (FDH).

Thyroid function and other laboratory results were shown in Table 1. The proband (III-4) again presented with significantly increased serum TT4 and TT3 and modestly increased FT4 and FT3 but nonsuppressed serum TSH in the latest test. TT4 showed about 2.2-fold increase above the upper limit of its normal range, while FT4 showed about 1.5-fold increase above its upper limit. Similar trends about TT4, TT3, FT4, FT3, and TSH were also found in her mother (II-3) and one of her uncles (II-2) as well as his son (III-2). Thyroid function parameters all fell into corresponding normal ranges in the eldest brother (II-1) and his son (III-1). Auto-antibodies of the thyroid including TGAb, TPOAb, and TRAb were all in normal ranges in all tested family members. Except a slight increase of ALT in the eldest brother (II-1), biochemical parameters including ALT, AST, ALB, and CREA were quite normal in all recruited members. The pedigree chart was then drawn according to the presentation of hyperthyroxinemia as shown in Figure 1. To exclude the laboratory bias of thyroid function, we retested all samples in the laboratory center of our hospital and verified that all the results were repeatable. We also investigated several local medical centers and commercial experimental corporations for additional assays of measuring thyroxines, but no substitute assays other than one- or two-step immunoassays are available at present.

**Figure 2:**
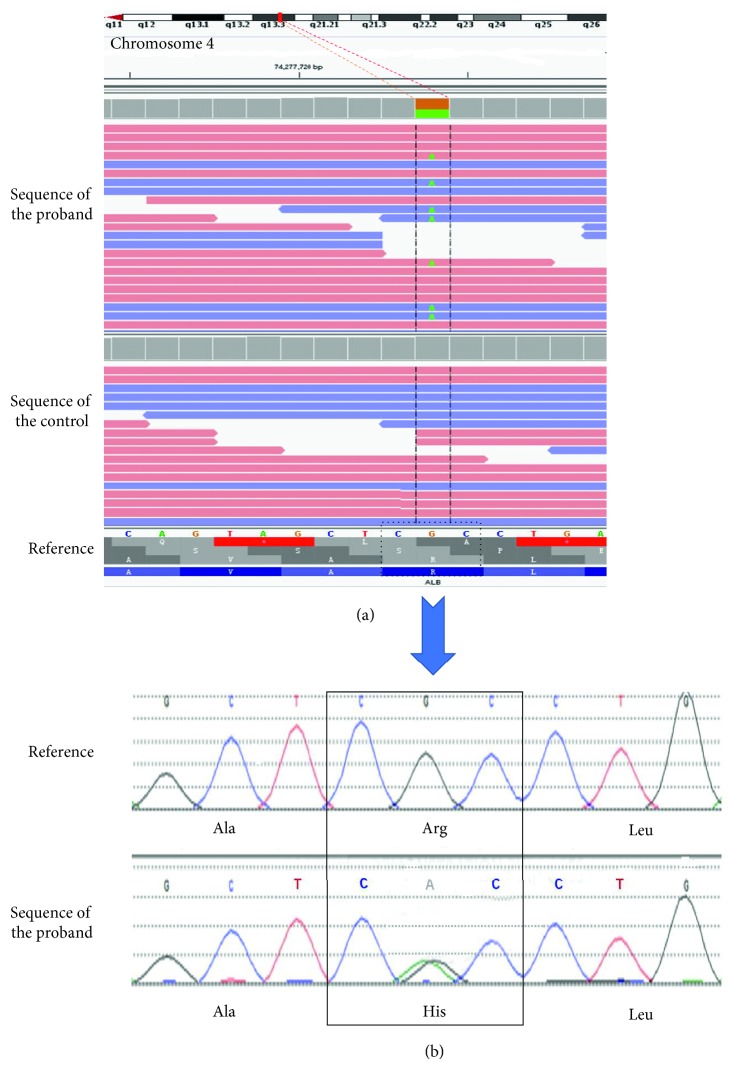
Gene sequencing of the proband. (a) Part of the genomic sequences on chromosome 4 within the human albumin gene compared with a normal control. The green point indicated the deviation from the reference genome at exon 7 (c.725G > A), and this codon mutation would replace arginine with a histidine in the peptide chain (R242H). (b) Confirmed results by the direct Sanger sequencing of the point mutation on the human albumin gene compared with the reference sequence. The codon CGC was substituted with CAC which further verified the R242H amino acid replacement. Ala: alanine, Arg: arginine, Leu: leucine, and His: histidine.

For the proband, the NGS technique including 4811 genes disclosed a heterozygous mutation at exon 7 (c.725G > A) on the albumin gene (Figure 2(a)), which would lead to arginine replacing by histidine after translation (R242H). The codon mutation was then further confirmed by the direct Sanger sequencing (Figure 2(b)). The same codon mutation was also specifically verified by the Sanger sequencing technique in her mother (II-3), her younger uncle (II-2), and the uncle's son (III-2). These were completely in agreement with their elevated serum TT4 and FT4. Negative mutation at the codon was shown in her eldest uncle (II-1), her cousins (III-1 and III-3), and her youngest sister (III-5), whose serum thyroid hormones were all normal in the latest serum test.

## 4. Discussion

We here report another Chinese family with FDH resulted from the codon mutation at exon 7 (c.725G > A) of the albumin gene, which in turn resulted in an amino acid replacement for arginine with histidine (R242H). The proband and other pedigrees with this heterozygous mutation presented with nearly 2.2-fold increase in serum TT4 and 1.5-fold increase in serum TT3. Simultaneously, all affected family members also showed about 1.5-fold increase in serum FT4 and mild increase in FT3 but nonsuppressed serum TSH. Increased serum FT4 had occasionally been the primary clue which promoted physicians to explore the underlying mechanism of hyperthyroxinemia in the proband. Clinically, we did not find any significant symptoms related to hyperthyroidism among all the affected pedigrees.

The R242H mutation (R218H without signal peptide included) for human albumin gene has been established as the most common cause of FDH among the reported cases [12–18]. In Chinese, totally four families with FDH has been reported until now [15–18], and R242H is the only cause for all families. No other codon mutations were found in Chinese as reported previously in other races [19–24]. Tang et al. [15] firstly confirmed the point mutation of the albumin gene in a 69-year-old male subject (strictly, not a family) who was accidentally found with hyperthyroxinemia. Except for the elevated serum TT4, the patient showed remarkably elevated FT4 in a one-step method but quite normal in another two-step laboratory assay. Tiu et al. [16] found the first family with R218H mutation in Hong Kong, serum TT4 and FT4 were all mildly increased in the proband and other affected pedigrees. But unfortunately, the proband's father underwent a long-term therapy with antithyroid drugs because of his presumed thyrotoxicosis. In the mainland China, Dai et al. [17] firstly described a FDH family with the same codon mutation in 2005. The proband, a 34-year-old male subject with palpitation and sweating, was found with elevated serum TT4 and quite normal FT4 and so were his mother and daughter. The proband was also treated with antithyroid drugs without any amelioration. Another FDH family from mainland China was reported recently by Wang et al. [18]. The proband of this family, a 61-year-old female, was diagnosed with hyperthyroidism on her complaint of palpitation and weakness as well as slightly elevated serum TT4 and TT3. Serum TSH was exactly in the normal range initially but significantly increased during a 2-year treatment with antithyroid drugs. Finally, she was confirmed with FDH and metastatic papillary thyroid cancer, which might be related to the elevated serum TSH [26]. As compared with the four reported families in Chinese [15–18], all affected members in the present family presented with significantly increased serum TT4 and nonsuppressed TSH and even mildly increased FT4 and FT3 in the one-step laboratory method. Most asymptomatic subjects may suffer from antithyroid treatment for this prominent character.

Serum concentration of FT4 in FDH patients varies according to different laboratory methods and immunodiagnostic systems. The one-step analog method for the FT4 measurement has been widely used by most laboratories for its high specificity and effectiveness [27]. But for patients with FDH, this method may lead to a high level of serum FT4 for increased binding affinity of the albumin to the labelled T4 analog during incubation [28]. On the other hand, one-step immunoassays by using immobilized T4 analog such as the Vitros system (Ortho Clinical Diagnostics) may make serum FT4 normal or even low [29]. The two-step method is thought to be an appropriate approach in FDH patients because almost all thyroxine-binding proteins are washed out before the labelled T4 analog penetrating into the incubation system, but indeed, unexpected high FT4 was also seen in FDH patients by using this assay method [30], especially in the Access system (Beckman Coulter) [28]. Ross et al. [31] found that the concentration of chloride in the incubation buffer also interferes with the final result either in the one-step or two-step method, and a chloride-free buffer may slightly decrease serum FT4 level while a high-chloride buffer may significantly increase it. To detect FT4 accurately, classical equilibrium dialysis may be taken into account to avoid these interferences from thyroxine-binding proteins [32] despite that the composition of the corresponding medium should also be investigated for FDH subjects [31]. Recently, Nagano et al. [6] established a gel filtration HPLC method which could evaluate the ability of serum T4 binding to HSA, and by using the technique, effects of HSA on T4 measurement would be accurately determined in any HSA mutant conditions. In addition, time-of-flight mass spectrometry has been recently adopted to exactly detect alteration of the HSA molecular mass [33]. We believed that FDH would be precisely diagnosed following on development of more and more specific laboratory methods. But first of all, physicians should take FDH into consideration when an asymptomatic patient presents with unusually elevated serum thyroxines, even if elevated FT4 is casually seen.

FDH should be excluded when several other diseases with elevated serum concentration of thyroxines such as RTH and TSH-secreting pituitary adenoma are in sought. For this aim, in the first place, clinicians should compare the results of thyroid function from different laboratory methods and platforms. The simple gene sequencing of the HSA gene may be further considered if necessary. As for the proband of the present family, we performed a direct gene sequencing of TR*β* to exclude RTH and pituitary MRI to rule out the TSH-secreting pituitary adenoma. It is obvious that both complicated and expensive techniques are unnecessary and not recommended if FDH was originally emphasized. Finally, significantly elevated serum TT4 is also common in diseases caused by other abnormal thyroxine-binding proteins, such as TBG and TBPA [8]. Serum concentration of both proteins should be measured if available.

In summary, we reported another Chinese family with FDH with the R242H mutation in the human albumin gene. All affected pedigrees in this family are characterized by elevated FT4 and FT3 in the one-step laboratory method but more remarkably high serum TT4 Clinical physicians should be aware of FDH when asymptomatic patients with hyperthyroxinemia and nonsuppressed serum TSH. Comparisons of results by using different assays and the further simple gene sequencing of the albumin gene may be helpful for excluding FDH. Antithyroid treatments must be recognized as contraindicated after the clear diagnosis of FDH.

## Figures and Tables

**Table 1:** Parameters of thyroid function and biochemical assays.

	Normal^*∗*^ ranges	II-1^+^	II-2	II-3	III-1	III-2	III-3	III-4^§^	III-5
Age (years)	—	44	41	39	17	12	8	11	5
TT4 (nmol/L)	66–181	132.4	**355.6** ^‡^	**321.7** ^‡^	122.6	**399.1** ^‡^	112.7	**391.4** ^‡^	138.3
TT3 (nmol/L)	1.3–3.1	2.3	**9.2** ^‡^	**7.0** ^‡^	1.9	**6.9** ^‡^	1.7	**8.9** ^‡^	2.5
FT4 (pmol/L)	12–22	15.27	**27.05** ^‡^	**29.10** ^‡^	15.06	**38.04** ^‡^	12.87	**32.08** ^‡^	17.19
FT3 (pmol/L)	3.1–6.8	3.56	**7.71** ^**‡**^	6.15	3.92	**8.12** ^‡^	3.39	**7.30** ^‡^	5.31
TSH (mIU/L)	0.27–4.2	1.87	2.61	2.38	3.40	3.33	2.54	1.34	2.99
TGAb (IU/L)	0–115	16.76	21.54	41.22	38.43	13.84	43.38	15.22	31.98
TPOAb (IU/L)	0–34	20.58	10.19	7.93	8.28	17.51	9.17	18.97	18.67
TRAb (IU/L)	0–1.58	<0.3	0.42	0.56	ND	<0.3	ND	<0.3	<0.3
ALT (U/L)	<45	**61** ^‡^	22	23	15	18	9	12	16
AST (U/L)	<45	37	17	19	13	25	11	16	13
ALB (g/L	34–53	41	39	44	43	45	41	43	43
CREA (*μ*mol/L)	62–115	65	100	78	63	48	56	67	55

^*∗*^Normal ranges of all parameters originate from the corresponding manufactures. ^+^The member labelled as no. 1 of the second generation (II) in the pedigree chart shown in Figure 1 and so on for other family members. ^§^The proband. ^‡^Elevated results deduced from corresponding normal ranges and so on for other laboratory results. ND: not detected. TT4: total thyroxine, TT3: total triiodothyronine, FT4: free thyroxine, FT3: free triiodothyronine, TSH: thyrotropin, TGAb: antithyroglobulin antibody, TPOAb: antithyroid peroxidase antibody, TRAb: anti-TSH receptor antibody, ALT: alanine aminotransferase, AST: aspartate aminotransferase, ALB: albumin, and CREA: creatinine.

## Data Availability

The data used to support the findings of this study are available from the corresponding author upon request.
